# Advanced Techniques for Skeletal Muscle Tissue Engineering and Regeneration

**DOI:** 10.3390/bioengineering7030099

**Published:** 2020-08-26

**Authors:** Moon Sung Kang, Seok Hyun Lee, Won Jung Park, Ji Eun Lee, Bongju Kim, Dong-Wook Han

**Affiliations:** 1 Department of Cogno-Mechatronics Engineering, College of Nanoscience and Nanotechnology, Pusan National University, Busan 46241, Korea; mskang7909@gmail.com; 2 Department of Optics and Mechatronics, College of Nanoscience and Nanotechnology, Pusan National University, Busan 46241, Korea; seokhyeon95@gmail.com (S.H.L.); dnjswjd158@gmail.com (W.J.P.); jelee7339@gmail.com (J.E.L.); 3 Dental Life Science Research Institute & Clinical Translational Research Center for Dental Science, Seoul National University Dental Hospital, Seoul 03080, Korea

**Keywords:** skeletal muscle, tissue engineering, tissue regeneration, electrospinning, 3D bioprinting

## Abstract

Tissue engineering has recently emerged as a novel strategy for the regeneration of damaged skeletal muscle tissues due to its ability to regenerate tissue. However, tissue engineering is challenging due to the need for state-of-the-art interdisciplinary studies involving material science, biochemistry, and mechanical engineering. For this reason, electrospinning and three-dimensional (3D) printing methods have been widely studied because they can insert embedded muscle cells into an extracellular-matrix-mimicking microenvironment, which helps the growth of seeded or laden cells and cell signals by modulating cell–cell interaction and cell–matrix interaction. In this mini review, the recent research trends in scaffold fabrication for skeletal muscle tissue regeneration using advanced techniques, such as electrospinning and 3D bioprinting, are summarized. In conclusion, the further development of skeletal muscle tissue engineering techniques may provide innovative results with clinical potential for skeletal muscle regeneration.

## 1. Introduction

**Figure 1:**
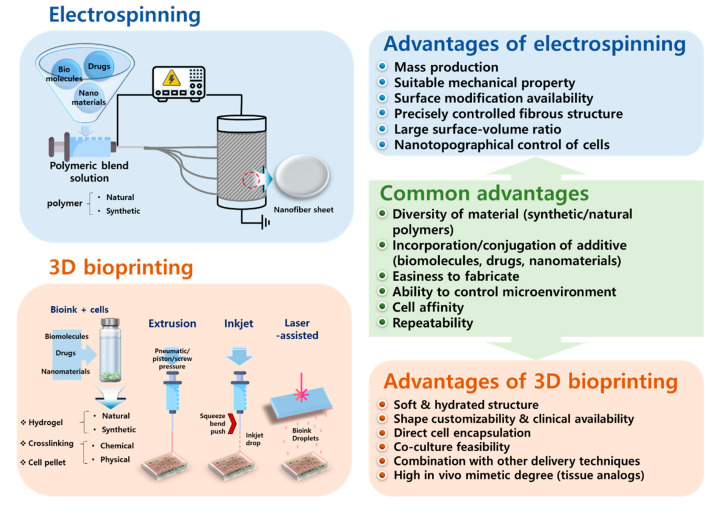
Relative comparison of technical advantages between electrospinning and 3D bioprinting to engineer skeletal muscle tissue constructs.

Skeletal muscle tissue has a highly differentiated and sophisticated microstructure that is often damaged by traumatic injury, tumor ablation, and degenerative disease, leading to muscle fiber atrophy. Despite the high regeneration capacity of skeletal muscle tissue, large volumes of muscle loss are not naturally recovered and need interventional support [1,2,3,4]. Therefore, several strategies, including surgery, medicine, physical therapy, cell therapy, nanotechnology, and tissue engineering, have emerged to promote skeletal muscle regeneration [5,6,7,8,9,10,11,12,13,14,15]. In particular, tissue engineering has been suggested as a novel approach to tissue regeneration due to its potential to regenerate tissue; however, state-of-the-art interdisciplinary studies are needed involving material science, cellular and molecular biology, and mechanical engineering. Scaffolds for muscle tissue engineering provide three-dimensional (3D) support that can help the growth of laden or seeded cells, an adequate microstructure, and cell signals by modulating cell–cell and cell–matrix interactions. In other words, scaffolds should have biofunctionality since the fate of the cells laden in the scaffold is determined by mechanical and biochemical signals from the scaffold. Therefore, recent studies have attempted to meet these requirements through the extensive recruitment of biomaterials and engineering methods. Electrospinning and 3D printing are representative techniques for skeletal muscle tissue engineering due to their feasibility of bulk but tailored microporous and aligned structure fabrication including wide material selection, ease of sample preparation, and a mild and simple process. Generally, the technical advantages of electrospinning and 3D printing for fabricating skeletal muscle tissue constructs have the potential to enhance the de novo formation of skeletal muscle (Figure 1). Both methods have their own specific strength, can be adapted according to the purpose of the study, and used complementarily for the engineering of skeletal muscle tissues. The biocompatibility and physicochemical properties of novel biomaterials have also been widely studied [15,16,17,18,19]. The transplantation of synthesized tissue analogues, the development of culture systems for maturation and functionalization, and the evaluation of the effectiveness of tissue analogues through animal and clinical trials are becoming key components of this research [20,21].

In this mini review, the recent research trends in scaffold fabrication for skeletal muscle tissue regeneration using advanced techniques, such as electrospinning and 3D bioprinting, are categorized (Table 1). Herein, we discuss recent technical progress in the skeletal muscle tissue engineering field, focused on the technical feasibility, material characteristics, and novel methods for overcoming current issues. We expect that this paper will provide a basis for research in the biomedical engineering and clinical fields.

## 2. Electrospinning

A nanofiber consists of tens to hundreds of nanometer-sized ultra-fine fibers possessing specific physicochemical characteristics. Nanofibers are characterized by a high surface-to-volume ratio and microporous structure. Nanofibers can be fabricated using various methods, such as melt-blown, flash-spinning, physical drawing, self-assembling, centrifugal spinning, and electrospinning [37,38,39,40,41]. Among these synthesis methods, electrospinning can control the size and morphology of nanofibers by adjusting the parameters, which allows these methods to be widely applied in the synthesis of tissue engineering scaffolds. Electrospinning can use almost every soluble polymer and additive and can fabricate various shapes and sizes with a wide range of physicochemical properties. The electrospinning process requires a simple and economical setup and can be modified by the addition of several molecules, processing, and technical parameters [42]. In particular, electrospun nanofibers have been receiving considerable attention in the field of tissue engineering because they provide a suitable microenvironment for cell behaviors, such as attachment, migration, proliferation, and differentiation [43,44]. Nanofiber scaffolds can mimic the morphological characteristics of an extracellular matrix (ECM), which has a microporous structure and can replicate and regenerate the environment of tissues in a three-dimensional state. Stem cells can sense the nanoscaled cues as myogenic differentiating factors [45]. Therefore, sophisticated nanotopographical control is important because it can spread biochemical signals to the cells. It is important to control the biofunctional moieties included in the nanofibers to provide proper signals to the loaded cells. Electrospinning can easily control the distribution and release of the biofunctional moieties within the nanofibers by controlling the morphological characteristics, polymer selection and modification, fiber diameter, etc. Properly treated electrospun nanofibers are suitable for implantation because they possess superior biodegradability and biocompatibility and are nontoxic and noninflammatory. Natural polymers exhibit superior biocompatibility, low immunogenicity, and a biofunctionality that allows them to control the skeletal muscle cell behaviors. The typical natural polymers include polysaccharide (cellulose, chitin, chitosan, and dextrose) and proteins (collagen, gelatin, fibrin, silk, and hyaluronic acid), DNA, and biopolymer derivatives [46]. Most natural polymers are biocompatible and bioactive; however, they exhibit low mechanical properties. Synthetic polymers, such as polycaprolactone (PCL), poly(methyl methacrylate) (PMMA), and poly(lactic-co-glycolic acid) (PLGA), have suitable mechanical properties and tailored biodegradability, but they are low in bioactivity. Therefore, most of the recent studies simultaneously incorporated natural and synthetic polymers to fabricate appropriate nanofibers with the proper physicochemical and biological properties.

The anisotropy of the tissue and matrix structure is the most peculiar aspect of skeletal muscles. In other words, skeletal muscle cells in the human body are embedded in the highly aligned ECM. The ability to mimic this aligned morphology leads to more realistic tissue models by replicating complex cell–matrix interactions in vivo. Soliman et al. synthesized highly aligned PCL/gelatin nanosheets using a manually prepared electrospinning system [22]. A porcine gelatin and PCL were dissolved in 1,1,1,3,3,3-hexafluoro-2-propanol (HFP) at a ratio of 7:3 in weight to provide nanofiber biocompatibility and structural stability. To evaluate the influence of the alignment of nanosheets on myoblasts, aligned and randomly oriented nanosheets were fabricated by a rotating drum and flat metal collector. The cultured myoblasts showed a high elongation, with nuclei and cytoskeleton aspect ratios of 20% and 150%, respectively. The myoblasts on the nanosheets showed high acetylcholine receptor (AChR) exhibition when cocultured with neurons. These results are of paramount importance as the muscle innervation and neuromuscular interaction should be studied to further our understanding of muscle cell behaviors. The prepared PCL/gelatin nanosheets could be used as a basic model for synaptogenesis and neuromuscular junction studies.

Many other studies developed micro-aligned scaffolds for muscle tissue engineering. Choi et al. also proved the importance of the alignment of nanosheets in inducing highly differentiated myotubes [23]. The authors fabricated poly(epsilon-caprolactone) (PCL)/collagen nanofiber meshes to induce myogenesis. Collagen and PCL were dissolved in HFP at a ratio of 1:1 in weight. Differently-oriented nanofibers were fabricated by collection on a rotating mandrel at various speeds. The morphogenesis of human skeletal muscle cells (hSkMCs) was controlled by aligning poly(epsilon-caprolactone)/collagen nanofiber meshes, resulting in enhanced adherence and proliferation compared with the randomly-oriented nanofibers. The degree of myotube maturation was evaluated by fluorescence-activated cell sorter (FACS) analysis and the length and alignment of the myotubes, which indicated that the aligned PCL/collagen aligned nanosheets considerably enhanced the maturation of hSkMCs. 

**Figure 2:**
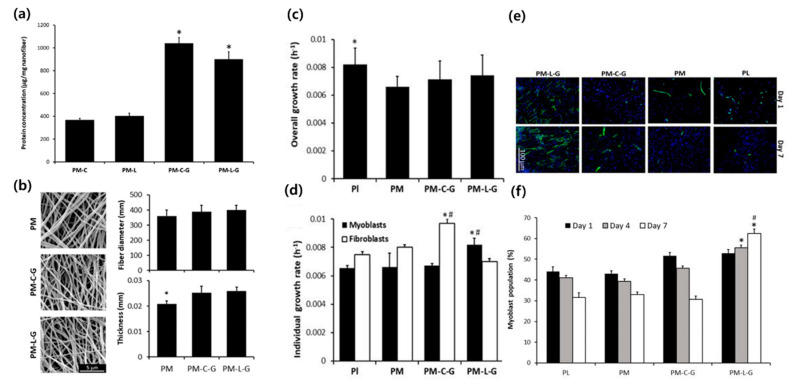
Comparison of the cell growth of the fibroblasts and myoblasts on each type of the nanofiber scaffolds. Abbreviations: Pl, PM, PM-C, PM-L, PM-C-G, and PM-L-G are the plastic surface, PMMA, PMMA-collagen, PMMA-laminin, PMMA-collagen-genipin, and PMMA-laminin-genipin. (**a**) Protein concentration. Genipin aided in collagen and laminin adsorption to nanofibers. (**b**) SEM image, diameter, and thickness of the electrospun nanofiber. (**c**,**d**) Overall and individual cell growth rate, respectively. PM-L-G enhanced the growth of myoblasts while it suppressed that of fibroblasts. (**e**) Fluorescence microscopy showing the myoblasts (green) and overall nucleus (blue). (**f**) Myoblast population within 7 days. Only PM-L-G showed a growing population of myoblasts. Reproduced from [24], copyright open access by MDPI 2017.

Still, several strategies are required to improve the in vivo functionality of skeletal muscle tissue scaffolds. Among the hurdles for successful implantation is fibroblast contamination, which leads to decreased growth of targeted muscle cells. Zahari et al. focused on the in vitro enrichment of isolated myoblasts to overcome the limitation induced by the contamination of fibroblasts (Figure 2) [24]. Isolated human muscle tissue is generally contaminated by fibroblasts, which grow faster than myoblasts in vitro. To engineer highly contractile muscle tissue with isolated muscle tissues, the degree of myoblasts has to be upregulated. The effects on collagen and laminin, which regulate the cellular population of myoblasts and fibroblasts, were compared [24]. Collagen or laminin was crosslinked to PMMA by a natural crosslinker, genipin. A PMMA solution was dissolved in hexafluoroisopropanol (HFIP) and electrospun on a rotating collector. PMMA nanosheets were coated with a laminin-1 and collagen type I solution in the presence of genipin. Cellular analysis indicated that the myoblast population significantly increased on the laminin-coated nanofiber scaffold in comparison with the collagen-coated one, suggesting the potential use of laminin in skeletal myoblast isolation.

The material mixture is often used to complement the physical characteristics of each polymer or biofunctional moiety. Bloise et al. prepared the ether-oxygen-containing electrospun microfibrous scaffolds with poly(butylene 1,4-cyclohexandicarboxylate-co-triethylene cyclohexanedicarboxylate) (P(BCE-TECE)) to engineer skeletal muscle tissue [25]. PBCE has good thermal stability and mechanical properties; however, it has low flexibility and biodegradability. To compensate for the disadvantages of PBCE, polyethylene glycol (PEG)-like moieties (TECE) were incorporated into PBCE polymers because they display remarkable flexibility and biodegradability. The electrospinning process was conducted under an optimized condition to fabricate fibers of each size (micro and submicro). The cellular study indicated that without biochemical cues, the mechanical and morphological characteristics of electrospun fibers are a crucial factor for determining cellular viability and functionality. C2C12 myoblasts showed enhanced cell adhesion, proliferation, and differentiation due to the aligned and high-TECE-containing fiber structures. The murine transplantation of the P(BCE-TECE) scaffold showed that it was highly vascularized and colonized by cells, indicating a perfect integration with the host muscle tissue. 

Similarly, Abarzúa-Illanes et al. used PCL/poly(ε-caprolactone) nanofibers decorated with decorin, a proteoglycan that induces myogenesis [26]. Decorin is known to not only upregulate the myogenic factors, such as follistatin, that promote differentiation and fusion of myotubes but also to downregulate myostatin, a myogenesis inhibitor. Decorin controls the myoblast differentiation by regulating transforming growth factor (TGF-β). Therefore, biofunctional cues such as decorin are often incorporated into the scaffold to effectively regulate the myogenic differentiation and maturation of skeletal muscle cells. In the study, PCL:PLGA dissolved in chloroform and decorin from bovine articular cartilage was added before and after electrospinning. Electrospinning was conducted under an optimized condition of the coverslips, dipped in a polymeric solution of an electrospinning source, on a copper-covered solid stainless-steel rotating cylinder. The authors compared the topological effect (alignment of nanosheets) and compositional characteristics (PLGA/decorin concentration) to the myogenesis of C2C12 myoblasts. The results indicated that both factors are related to myogenesis; however, at a certain concentration range, higher PLGA and decorin concentrations induced myogenesis more than the fiber alignment.

PLGA is a widely used synthetic polymer due to its biodegradability and biocompatibility. To address the functional drawbacks of PLGA-based nanofibers, functional materials can be decorated. Shin et al. synthesized an Arg-Gly-Asp (RGD) peptide displaying M13 bacteriophage and graphene oxide (GO) into PLGA nanofibers to facilitate the myogenesis of C2C12 myoblasts [27]. The RGD peptide and GO were operated as adhesion and myogenesis stimulating cues, respectively. Bacteriophages cannot infect and replicate in human cells, so they are a human-safe virus that are potentially applicable in the biomedical field [47,48,49]. GO is among the biofunctional 2D nanomaterials that were reported to possess the ability to stimulate the differentiation of myoblasts. PLGA was dissolved in HFIP, and an RGD-M13 phage was suspended in tris-buffered saline (TBS) buffer and blended with the PLGA solution. Randomly-oriented nanosheets were fabricated to exclude the topological effects. C2C12 myoblasts were favorably adhered and proliferated on the prepared nanofibers. A highly mature myotube was induced by incorporating the RGD-M13 bacteriophage. The results suggested that the incorporation of biofunctional cues such as RGD-M13 bacteriophages and GO can enhance the biofunctionality of PLGA-based nanofiber and the potential application of GO in the biomedical field.

The premyogenic effect of human adipose-derived stem cells (ASCs) indicates their promise as a cell source for skeletal muscle regeneration. Gilbert-Honick et al. used fibrin-based uniaxially-aligned fibrin hydrogel electrospun fibers for muscle reconstruction using ASCs [28]. Fibrinogen and sodium alginate solutions were electrospun, and polyethylene oxide was added to each solution to increase the viscosity during electrospinning. After electrospinning, C2Cl2 cells were seeded and thrombin was added as crosslinking agents. Despite low in vitro myogenic potential, electrospun fibrin fibers combined with ASCs promoted moderate muscle reconstruction ASC coseeded fibers, which exhibited the regenerating muscle marker, embryonic myosin, and the mature muscle marker, myosin heavy chain, after one and three months of transplantation, respectively, suggesting the myogenic differentiation-inducing capability of ASCs with C2C12 cells.

Oxidative stress is important to the fate of myoblasts because oxidative stress is associated with pathological conditions, such as myotonic dystrophy. To enhance the electroactivity and antioxidant properties of nanosheets, Manchineella et al. introduced pigmented silk nanosheets as a skeletal muscle tissue engineering scaffold [29]. Silk fibroin was extracted from mulberry silkworm *Bombyx mori* cocoons and refined. In addition, silk fibroin and melanin solutions were reconstituted from HFIP and electrospun in a conventional stainless-steel collector, then treated with methanol to increase the aqueous stability through β-sheet formation in silk. The prepared nanosheets showed high biocompatibility and biodegradability and did not cause oxidative stress. Silk and melanin successfully acted as electroconductive cues, which led to enhanced cellular signaling. The composite electrospun scaffold finally promoted myogenic differentiation, showing the advantages of adopting a silk-based material in skeletal muscle tissue engineering.

## 3. Three-Dimensional Bioprinting

**Figure 3:**
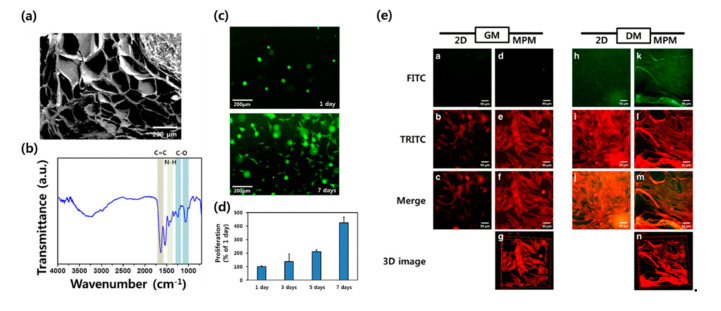
GHPA hydrogel as a 3D printable bioink for myogenic differentiation. (**a**) SEM images and (**b**) FTIR graph of the prepared GHPA hydrogel. (**c**) Cell viability for 1 day (top) and 7 days (bottom) of cultured myoblasts in the growth medium. (**d**) Cell proliferation assessed by a CCK-8 assay. (**e**) Multiphoton fluorescence microscopy indicating MHC (green) and F-actin (red). Results indicated that the myoblasts were successfully differentiated in the GHPA hydrogels. Reproduced from [65], copyright open access by Creative Commons License Deed.

Three-dimensional bioprinting is an additive manufacturing technology that fabricates tissue analogues by stacking living cells and biomaterials layer by layer. Three-dimensional bioprinting is emerging as a powerful tool for tissue engineering because it can easily fabricate bulk and sophisticated tissue, mimicking the structure and endowing cells with a biomimetic 3D microenvironment culture in the desired shape. Various 3D bioprinting methods, including inkjet, extrusion, and laser-assisted methods, are widely used because they ensure high cell viability [30,50,51,52,53,54,55,56]. However, these methods have their limitations. In the case of the inkjet method, the resolution of the printed constructs may be limited because of the nozzle-injection style and material characteristics [57]. With the extrusion method, it is difficult to construct large free-form tissue structures due to inadequate structural integrity, mechanical stability, and printability [58]. The laser-assisted method requires the rapid gelation of hydrogels to achieve high-resolution printed patterns, resulting in low flow rates [59]. Hydrogel is a three-dimensional hydrophilic polymer network structurs, in which more than 90% of the components are composed of water. The hydrogel can be applied as a skeletal tissue engineering scaffold due to its tissue-like characteristics, such as high water content, microporous structure, soft mechanical properties, and biocompatibility [60,61,62]. Hydrogels can be divided into chemical hydrogels and physical hydrogels depending on the crosslinking method. When hydrogel polymer chains form a network structure by covalent bonding, it is called a chemical hydrogel (also known as a chemically crosslinked hydrogel), and when it is formed by secondary bonding, such as hydrogen bonding, ionic bonding, hydrophobic interaction, and electrostatic interaction, it is called a physical hydrogel (also known as a physically crosslinked hydrogel). Recently, several strategies have emerged to reduce cytotoxicity, especially using the chemical crosslinking method, by promoting a mild reaction condition [63,64,65]. Physical hydrogels are also widely studied due to the suitability of the environment of physical hydrogels for the encapsulation of fragile substances, such as cells, because physical hydrogels are not crosslinked by potentially toxic chemical additives [65]. Kim et al. used gelatin hydroxyphenylpropionic acid (GHPA) hydrogel as bioink for skeletal muscle tissue engineering (Figure 3) [65]. A gelatin solution was reacted with 1-ethyl-3-(3-dimethylaminopropyl)carbodiimide hydrochloride/N-hydroxysuccinimide (EDC/NHS), and an HPA solution was added to synthesize a GHPA hydrogel. The prepared hydrogel can be crosslinked in situ in a mild reaction condition using horseradish peroxidase and hydrogen peroxide (H_2_O_2_), which leads to a high printing fidelity and cell viability. The printed construct was cultured in a myogenic differentiation medium and showed an elongated myotube with MHC expression, which was evaluated by multiphoton microscopy.

Recently, novel strategies in both printing technology and materials have been suggested. A sophisticated and multimodal injection system, the systematic development of a 3D printing module, and a sacrificial mold to create a more stable structure have been suggested [36,66,67,68,69]. The proper selection of a bioink is among the most important factors ensuring successful 3D bioprinting. Bioink is a printing material for 3D bioprinting that needs to possess adequate physicochemical and biocompatible properties before, during, and after 3D printing to achieve a high shape fidelity and cell viability. Bioink should not hinder the nutrient support, cell–cell or cell–matrix interactions, or the removal of cell metabolites during cell culturing. An aligned microenvironment is known to promote myogenesis by enhancing the behavior of muscle cells because it can mimic the biological and mechanical stimulus of natural ECM. Therefore, many studies focused on the development of a bioink that reproduces the aligned microstructure of ECM [70,71,72].

Among the limitations of 3D bioprinting is its low resolution that cannot perfectly mimic the natural microstructures. To overcome this issue, the microfluidic printing system was developed, which enabled improved fidelity compared with the conventional extrusion methods. In this method, multiple materials and cells can be deposited in the desired position to allow recapitulation of the whole muscle histoarchitecture. Costantini et al. presented a strategy for the fabrication of artificial skeletal muscle tissue based on a microfluidic printing head coupled to a co-axial needle extruder, which enabled a highly fine deposition of cell-laden hydrogel fibers [57]. The prepared bioink consists of PEG-fibrinogen biopolymer fibers that feature the photocurability and encapsulation of laden cells within aligned hydrogel fibers. Fibrinogen molecules were first cleaved into smaller fragments in a formic acid solution, containing cyanogen bromide. In addition, the fragmented fibrinogen was treated with tris(2-carboxyethyl) phosphine hydrochloride (TCEP) to reduce disulfide bonds to the thiol group and then reacted with PEG-diacrylate. To prepare the bioink, sodium alginate and PEG-fibrinogen were mixed. Alginate acted as a temporary templating material to enhance the fidelity of the fibers and allow for crosslinking with C2Cl2, whereas PEG-fibrinogen generated the matrix in which embedded myoblasts could grow and differentiate. After 3D bioprinting, C2C12 myoblast-laden constructs showed a high degree of viability and alignment. After 3–5 days of culture, the encapsulated C2C12 myoblasts started fusing and forming multinucleated myotubes, indicating the myogenic inducement of bioink. The prepared bioink supported high viability of laden C2C12 myoblasts. Contractility, myotube formation and maturation were spontaneously induced by laden biological cues. Furthermore, agrin, a proteoglycan responsible for neuromuscular junctionformation, was preserved, and AChR clusters were observed in the 3D-printed muscle construct. These results suggested that this sophisticated printing technique contributes to the maturation of printed skeletal muscle tissues.

Natural polymers have many advantages as bioinks due to their biochemical properties; however, most of them lack the mechanical properties and hardness to be printed into fine constructs. To optimize the mechanical characteristics of natural polymer bioink, crosslinkable synthetic polymers have been incorporated. Mozetic et al. presented a 3D bioprinting methodology using a pluronic/alginate hydrogel for C2C12 myoblast engineering [37]. To maintain cell integrity and viability during the printing process, viscosity was optimized combining the natural polymer alginate and synthetic polymer pluronic F127. Pluronic F127 provided an ECM-like multiporous environment to the cells, whereas alginate provided the chemical crosslinking property by calcium incorporation. Sodium alginate was dissolved in culture media, and pluronic F127 was then dissolved into the solution. The cell-laden pluronic/alginate hydrogel was extruded using a custom-designed 3D printer and a pneumatic dispensing syringe. Aligned parallel fibers were fabricated and crosslinked by C2Cl2. The thermoresponsive behavior of pluronic allows for suitable 3D printing fidelity, and the crosslinking property was endowed with alginate. The printed constructs were cultured and showed an elongated cytoskeleton and nucleus morphology of laden cells. These properties finally led to the myogenesis of embedded myoblasts, which was confirmed by the enhanced expression of myogenic differentiation markers, such as myogenin, α-sarcomeric actin, and MyoD. The 3D-printed construct confined cells into aligned printed fiber that can produce many small muscle bundles in which myotubes are already aligned along the fiber direction.

As mentioned in the electrospinning section, the aligned internal structure of the skeletal muscle scaffolds supports the growth and maturation of cells. In 3D-bioprinting-based technology, the fabrication of aligned microstructures has been introduced. A hydrogel using self-assembly can create an aligned microenvironment that induces muscle cells to grow anisotropically. Arab et al. prepared nanofibrous self-assembling peptide hydrogels for the 3D bioprinting of skeletal muscle tissues [32]. The prepared CH-01 and CH-02 tetrameric self-assembling peptides showed a nanosized fibrous intrastructure that was significantly smaller than that of bovine collagens. Accordingly, 3D-bioprinted C2C12 myoblasts showed an increased fusion index and nuclear aspect ratio compared to controls (TCP and Matrigel). These results also implied that the ability to induce the alignment of muscle tissue is highly correlated with muscle tissue differentiation and maturity. Kim et al. presented a preculture of cell-laden bioink for the inducement of highly-aligned and matured myotubes after 3D bioprinting [33]. The authors used gelatin methacryloyl (GelMA)-based hydrogel and a precultured C2C12 myoblast-loaded hydrogel until an anisotropic morphologically-shaped cell/cytoskeleton was developed. The results indicated that the precultured bioink/cell showed an enhanced alignment and maturation of laden cells compared to the general cell-laden bioink.

García-Lizarribar et al. introduced a GelMa-based composite hydrogel for the 3D bioprinting of functional skeletal muscle tissues [34]. GelMA is methacrylic anhydride-functionalized gelatin hydrogel endowed with a photopolymerizable property. Although it has high biocompatibility and mild crosslinking property, GelMA lacks versatility in terms of its mechanical properties and has a poor degradation resistance. In the study [34], polysaccharides, such as alginate and carboxymethyl cellulose (CMC), were incorporated into GelMA, and different formulations, such as poly(ethylene glycol) diacrylate-added GelMA, were compared. GelMA, CMC, and alginate were dissolved in phosphate-buffered saline (PBS) and methacrylated and prepared as GelMA-CMCMA and GelMA-AlgMA. In addition, cell-laden hydrogels were 3D-bioprinted in aligned fibers with 200 µm nozzles. GelMA-CMCMA and GelMA-AlgMA exhibited increased degradation resistance and maintained their 3D structure in the culture environment, unlike GelMA-PEGDA. C2C12 myoblasts did not hinder their growth and showed enhanced cellular behavior viability, proliferation, and alignment, indicating the potential use of GelMA and CMCMA as bioinks for skeletal muscle tissue engineering.

Decellularization is another method to reproduce the microenvironment of skeletal muscle tissue. Most hydrogels, such as collagen, fibrin, and alginate, fail to perfectly mimic the structural, biochemical, and mechanical complexity of the ECM and do not produce the cellular morphology and behaviors of native tissue. Cells should be ideally surrounded by a native ECM environment; therefore, several methods have been introduced to decellularize the ECM and prepare bioink. The decellularized ECM (dECM) from skeletal muscle tissue also possesses several myogenic-inducing factors and can be used as biochemical stimulating factors in skeletal muscle tissue engineering. Kim et al. used dECM/PVA fibrillated bioink modified by the methacrylate process to produce a mechanically stable, uniaxially aligned in situ, and microtopographical intrastructural bioink [35]. dECM-MA was dissolved and mixed with PVA, and 3 wt% dECM-MA was dissolved in PBS to prepare the bioink. The prepared dECM-MA/PVA bioink was 3D-printed with a pneumatic dispenser using a 150 µm nozzle, then crosslinked by UV radiation. The results indicated that the topographical cues induced by PVA fibers induced the alignment of the C2C12 myoblasts, and the dECM component led to the highly-matured myogenic differentiation of cells. Choi et al. introduced skeletal-muscle-tissue-derived dECM)bioinks possessing native ECM components, such as collagen, glycosaminoglycans (GAGs), and growth factors [36]. The porcine tibialis anterior muscle was collected and decellularized by a general protocol [73]. The extracted muscle tissues were modified as follows: minced to 1 mm in thickness; treated with sodium dodecyl sulfate (SDS) to eliminate the remaining cells; treated with Triton-X and DNAse; treated with isopropanol for the removal of lipids; disinfected; and finally, washed, lyophilized, and milled to obtain a fine dECM powder. Three-dimensional tissue analogues were printed by a custom-designed 3D bioprinter with a PCL geometrical constraint to induce alignment. Three-dimensional bioprinted constructs supported high cell viability and contractility as well as myotube differentiation and maturation, suggesting the specific effect of dECM bioink for mimicking the structural and functional properties of the native ECM of skeletal muscle tissues.

## 4. Conclusions

This review summarized the recent progress in skeletal muscle tissue engineering techniques, focusing on electrospinning and 3D bioprinting. Electrospinning and 3D bioprinting are the most attractive techniques in the field of developing skeletal tissue engineering due to their potential to fabricate ECM-reproducing scaffolds that provide several clinical benefits. The biofunctionality and physicochemical characteristics, depending on the material characteristics and fabrication methods, were discussed. From the results described herein, the fabrication fidelity, mechanical stability, biocompatibility, and myogenic-inducing properties have been significantly increased by introducing several novel strategies. 

Several other approaches are possible to achieve clinically applicable treatments of muscle injuries such as understanding the underlying mechanisms of muscle loss or repair, exploration of a new drug, and evaluation of biocompatibility or biofunctionality of materials. [74,75] To improve scaffold- or cell-based intervention, Sicherer et al. directly compared different muscle loss models [76]. Here, we elucidated the advantages and characteristics of engineering techniques of skeletal muscle tissue focused on the fabrication method, material properties, and evaluation methods. Based on this multidisciplinary research, advancing engineering techniques can achieve skeletal muscle tissue regeneration.

In conclusion, the results presented in this review show that tissue engineering in skeletal muscle regeneration has promising prospects. Although recent engineering techniques have some limitations that need to be overcome by future research, we expect that the further development of tissue engineering will provide innovative results with clinical potential for novel skeletal muscle regeneration.

## Figures and Tables

**Table 1:** Recent research on skeletal muscle tissue engineering enabled by electrospinning and 3D bioprinting.

Fabrication Technique	Material	Cell	Experimental Method	Fundamental Novelty	Reference
Electrospinning	PLC/gelatin	NG108-15, C2C12	In vitro	Neuromuscular interaction	[22]
	PCL/collagen	hSkMC	In vitro	Nanofiber alignment	[23]
	Laminin/PMMA	Extracted human skeletal muscle cell	Ex vivo	Fibroblast contamination prevention	[24]
	P(BCE-TECE)	C2C12	In vitro and in vivo	Suitable mechanical property by the mixture of polymers	[25]
	PCL/PLGL-decorin	C2C12	In vitro	Myogenic inducing effect of decorin	[26]
	PLGA-RGD-GO	C2C12	In vitro	Myogenic inducing effect of GO	[27]
	Fibrinogen/alginate	ASC, C2C12	In vivo and ex vivo	ASC–C2C12 cell–cell interaction	[28]
	Silk fibrinogen, Silk/melanin	C2C12	In vitro	Electroactive and antioxidant nanofiber	[29]
3D bioprinting	PEG-fibrinogen	C2C12	In vitro and in vivo	Co-axial needle extruder for high fidelity	[30]
	Pluronic/alginate	C2C12	In vitro	Microfabrication with aligned deposition	[31]
	CH-01 and CH-02 tetrameric self-assembling peptides	C2C12	In vitro	Nanofibrous self-assembling peptide for aligned microenvironment	[32]
	GelMA	C2C12	In vitro	Preculture of cell-laden bioink	[33]
	GelMA-Alginate/CMC	C2C12	In vitro	Mechanical stabilization of GelMA-based hydrogel	[34]
	Methacrylated dECM/PVA fibrillated	C2C12	In vitro	Decelluarization, internal fibrous structure	[35]
	dECM	C2C12	In vitro	Decelluarization	[36]
